# ASS1 and ASL suppress growth in clear cell renal cell carcinoma via altered nitrogen metabolism

**DOI:** 10.1186/s40170-021-00271-8

**Published:** 2021-12-03

**Authors:** Sanika Khare, Laura C. Kim, Graham Lobel, Paschalis-Thomas Doulias, Harry Ischiropoulos, Itzhak Nissim, Brian Keith, M. Celeste Simon

**Affiliations:** 1 Abramson Family Cancer Research Institute, Perelman School of Medicine, University of Pennsylvania, Philadelphia, PA 19104 USA; 2 Children’s Hospital of Philadelphia Research Institute and Departments of Pediatrics and Pharmacology, Perelman School of Medicine at the University of Pennsylvania, Philadelphia, PA 19104 USA; 3 Division of Genetics and Metabolism, Children’s Hospital of Philadelphia, Philadelphia, PA 19104 USA; 4 Department of Pediatrics, Biochemistry, and Biophysics, University of Pennsylvania, Philadelphia, PA 19104 USA; 5 The Wistar Institute, Philadelphia, PA 19104 USA

**Keywords:** ccRCC, Urea cycle, Argininosuccinate synthase 1, Argininosuccinate lyase, Aspartate, DNA synthesis, Nitric oxide metabolism

## Abstract

**Background:**

Kidney cancer is a common adult malignancy in the USA. Clear cell renal cell carcinoma (ccRCC), the predominant subtype of kidney cancer, is characterized by widespread metabolic changes. Urea metabolism is one such altered pathway in ccRCC. The aim of this study was to elucidate the contributions of urea cycle enzymes, argininosuccinate synthase 1 (ASS1), and argininosuccinate lyase (ASL) towards ccRCC progression.

**Methods:**

We employed a combination of computational, genetic, and metabolomic tools along with in vivo animal models to establish a tumor-suppressive role for ASS1 and ASL in ccRCC.

**Results:**

We show that the mRNA and protein expression of urea cycle enzymes ASS1 and ASL are reduced in ccRCC tumors when compared to the normal kidney. Furthermore, the loss of ASL in HK-2 cells (immortalized renal epithelial cells) promotes growth in 2D and 3D growth assays, while combined re-expression of ASS1 and ASL in ccRCC cell lines suppresses growth in 2D, 3D, and in vivo xenograft models. We establish that this suppression is dependent on their enzymatic activity. Finally, we demonstrate that conservation of cellular aspartate, regulation of nitric oxide synthesis, and pyrimidine production play pivotal roles in ASS1+ASL-mediated growth suppression in ccRCC.

**Conclusions:**

ccRCC tumors downregulate the components of the urea cycle including the enzymes argininosuccinate synthase 1 (ASS1) and argininosuccinate lyase (ASL). These cytosolic enzymes lie at a critical metabolic hub in the cell and are involved in aspartate catabolism and arginine and nitric oxide biosynthesis. Loss of ASS1 and ASL helps cells redirect aspartate towards pyrimidine synthesis and support enhanced proliferation. Additionally, reduced levels of ASS1 and ASL might help regulate nitric oxide (NO) generation and mitigate its cytotoxic effects. Overall, our work adds to the understanding of urea cycle enzymes in a context-independent of ureagenesis, their role in ccRCC progression, and uncovers novel potential metabolic vulnerabilities in ccRCC.

**Supplementary Information:**

The online version contains supplementary material available at 10.1186/s40170-021-00271-8.

## Background

Substantial metabolic adaptations are now regarded as a hallmark of malignant transformation [1, 2], where both hematological and solid malignancies exhibit significant phenotypic differences from their normal tissues of origin. Studying altered cancer cell metabolism is not limited to evaluating a single event; rather, it is akin to observing changes in a complex and wide network of genes, enzymes, metabolites, and microenvironments [3, 4]. With their consistent and extensive metabolic rewiring, clear cell renal cell carcinomas (ccRCCs) represent an excellent model system to study the altered metabolism in cancer [4–9].

ccRCC tumors constitute the most common subtype of all renal cancers [10], accounting for > 75% of diagnoses. Genetic and pathological analyses have revealed that aberrant metabolism is a defining feature of this human disease. ccRCCs derive their “clear cell” name based on their histological appearance. Grossly, they appear yellowish with necrosis and hemorrhage, and histologically, ccRCC samples consist of thin-walled cells filled with abundant lipids and glycogen—giving the “clear cell” appearance. More than 90% of ccRCC tumors exhibit chromosome 3p aberrations, where one copy of the von Hippel Lindau (*VHL*) gene is ubiquitously lost and the remaining allele mutated or silenced [11–14]. *VHL* loss leads to downstream stabilization and constitutive activation of hypoxia-inducible factors (HIFs), which in turn drive malignant transformation in ccRCC. HIFα activation enhances the transcription of genes involved in angiogenesis (*VEGFA*), glucose uptake (*GLUT1*), survival (*survivin*), migration and invasion (*CXCR4*), and proliferation (*EGFR*) [15, 16].

**Fig. 1:**
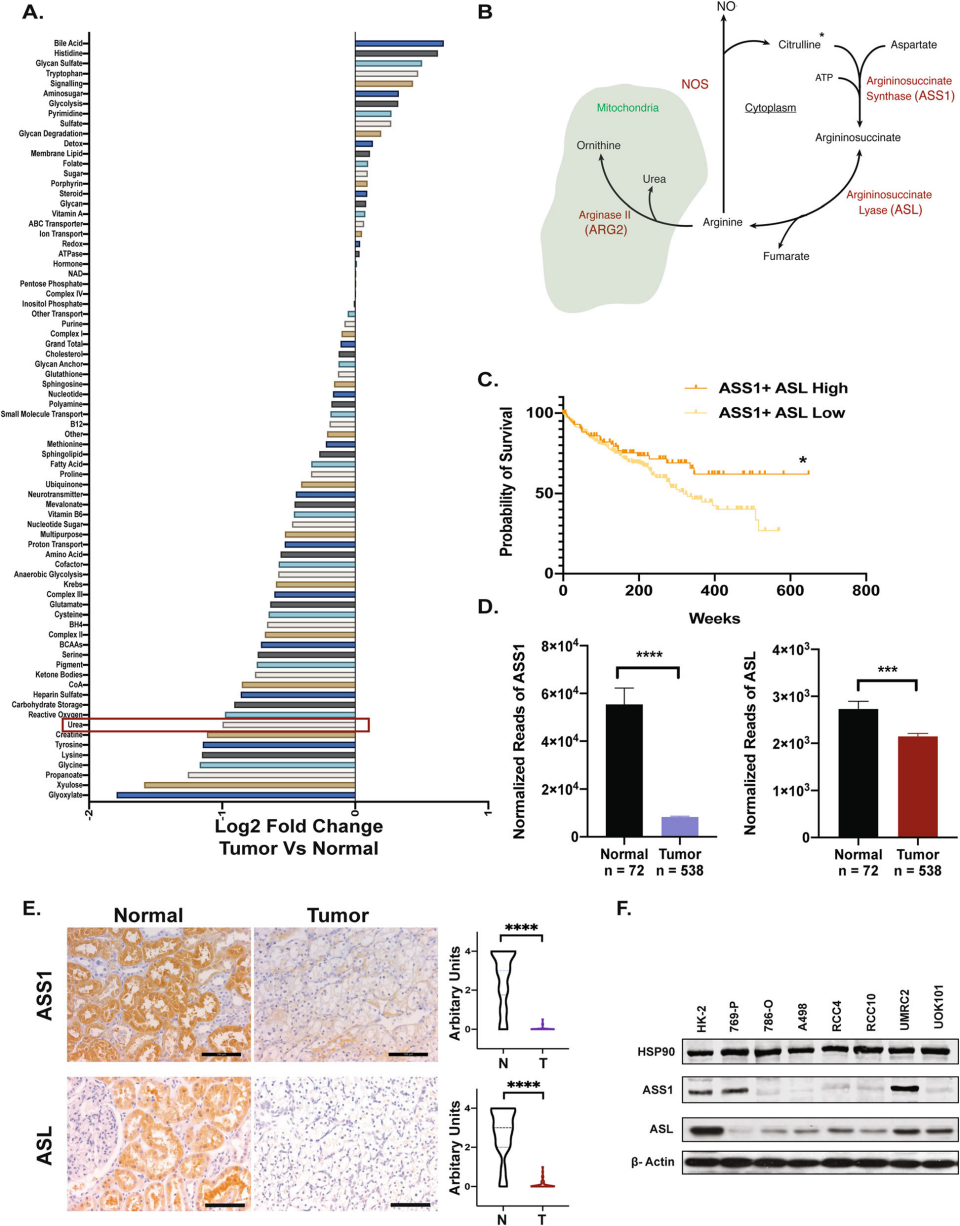
ccRCC tumors exhibit alterations in urea cycle components. **A** Metabolic gene set analysis of mRNA-seq data from The Cancer Genome Atlas (TCGA) classified based on KEGG. A ranked list of metabolic gene sets was generated based on log2 scale fold expression changes in ccRCC tumors (*n* = 538) and normal kidney (*n* = 72). **B** The urea shunt as configured in the kidney. *Citrulline from diet. **C** Survival analysis based on TCGA expression data for ASS1 and ASL high (*n* = 90) and ASS1 and ASL low (*n* = 236) patients. *p < 0.05. **D** mRNA-seq data of ASS1 and ASL in normal kidney vs ccRCC tumors (TCGA). ****p < 0.001, ***p = 0.001, Mann-Whitney test. **E** Representative immunohistochemistry images with quantification for ASS1 or ASL protein expression in matched tumor-normal pairs (Biomax KD601) *n* = 30. Scale bars represent 100 μm. ****p < 0.001, Welch’s *t*-test. **F** ASS1 and ASL protein levels in HK-2 (immortalized kidney epithelial) cells and various ccRCC cell lines. HSP90 and actin serve as loading controls

The VHL-HIF axis is not only central to ccRCC tumorigenesis but also contributes towards their rewired metabolism, including consistent elevated rates of glycolysis without glucose oxidation based on intraoperative labeling in ccRCC patients [17]. Other metabolic changes include altered ammonia metabolism with multiple urea cycle enzymes significantly underexpressed [6, 18]. The hepatic urea cycle is an essential detoxification mechanism that converts ammonia generated from protein turnover into urea [19]. However, the traditional cycle exists more as a “urea shunt” in the kidney (see Fig. 1B). In normal human physiology, the kidneys excrete large quantities of urea and produce arginine for export to other organs. Altered expression of urea cycle enzymes in multiple cancers reduces nitrogen waste generation while simultaneously redirecting carbon and nitrogen to anabolic biomass generation [19–21]. There is also evidence of ammonia instead being redirected towards amino acid synthesis (e.g., glutamine and glutamate) in breast cancer [22], obviating the need for an intracellular urea cycle. These amino acids are pivotal in cancer cell growth, as they serve as precursors for biosynthesis of nucleotides, lipids, amino acids, and antioxidants like glutathione [22], and maintain TCA anaplerosis. Dysregulation of urea cycle components can contribute towards enhanced glutamine utilization, a hallmark of numerous cancer cells, especially in hypoxic tumor microenvironments [3].

Urea cycle enzymes, including nitric oxide synthase, convert arginine to citrulline, which is then converted to argininosuccinate by argininosuccinate synthase 1 (ASS1), fumarate and arginine by argininosuccinate lyase (ASL), and ultimately catabolized to urea and ornithine by arginase 2 (ARG2). The expression of ARG2, a mitochondrial urea cycle enzyme in the kidney, is consistently downregulated in ccRCC tumors when compared to healthy tissue [18]. Reduced ARG2 levels promote tumor growth by two key mechanisms, i.e., conserving pyridoxal-5′-phosphate, a critical biosynthetic cofactor, and preventing a toxic buildup of polyamines [18].

Aspartate, a urea cycle intermediate, is also a key metabolite in proliferating cells [19]. Cells import only minuscule amounts from the microenvironment and instead depend on de novo synthesis for their aspartate. Intracellular synthesis of aspartate requires a functional TCA cycle, electron transport chain (ETC), and healthy mitochondria [23, 24]. Cancer cells often have suboptimal TCA cycle and ETC function and thus have limited intracellular pools of aspartate. Aspartate is transported from the mitochondria to the cytosol via citrin. Once in the cytosol, it broadly has two key metabolic fates—conversion to argininosuccinate (by ASS1) or orotic acid (by the CAD complex). In line with the enhanced pyrimidine synthesis, proliferating cells upregulate citrin expression and CAD activity, while simultaneously downregulating ASS1 expression [20–25]. Thus, ASS1 downregulation supports proliferation by shunting aspartate towards pyrimidine synthesis. However, this can result in another metabolic vulnerability: cells lacking ASS1 (and ASL) are arginine auxotrophs and depend on exogenous sources for their intracellular supplies. As a result, arginine deprivation has emerged as an attractive therapy in cancers lacking ASS1 (and ASL) [26, 27].

Interestingly, tumors lacking ASS1 like thyroid, liver, and kidney exhibit an imbalance in their pyrimidine/purine ratios, which in turn leads to novel surface protein signatures [28]. Recent studies indicate that such tumors are exquisitely sensitive to immune checkpoint therapy. Regulation of *ASS1* expression is complex, and diverse factors including MYC, p53, and HIF1-α are reportedly involved [28, 29]. However, *ASS1* is also over-expressed in a few other malignancies. High ASS1 levels in colon cancer are thought to support arginine synthesis and help the cells survive low-nutrient environments [30]. The role of ASL expression in carcinogenesis is more ambivalent. High *ASL* expression is considered a poor prognostic factor in breast, liver, and colorectal cancers [31]. Downregulation of ASL activity is associated with lowered arginine and nitric oxide synthesis, which can regulate the immune microenvironment and bolster tumor growth in a context-dependent manner [32–35]. Interestingly, the reaction catalyzed by ASL is reversible in nature and has been reported to convert fumarate to argininosuccinate in certain renal tumors [36].

In this study, we establish widespread loss of urea cycle enzymes ASS1 and ASL in ccRCC patients. We further elucidate the contributions of ASS1 and ASL loss towards ccRCC progression by knocking down the genes in normal renal cells and re-expressing them in ccRCC cells. We observed significant growth suppression with the combined re-expression of ASS1 and ASL in ccRCC cells. Furthermore, we show that ASS1 and ASL expression regulates cellular aspartate levels, nucleotide synthesis, and nitric oxide production. Taken together, our data suggests that ASS1 and ASL act as potential metabolic tumor suppressors in ccRCC, and their loss conserves cellular aspartate pools and regulates nitric oxide generation to give ccRCC cells a proliferative edge.

## Methods

### Reagents

**Table:** 

Reagent or resource	Source	Identifier
**Antibodies**		
β-Actin	Santa Cruz	sc47778
ASS1	Abcam	ab124465
ASS1	Santa Cruz	s c-99178
ASL	Sigma-Aldrich	HPA016646
CD31	Abcam	ab28364
Ki67	BD Biosciences	#550609
β-Tubulin	Cell Signaling	#2146
p21	Cell Signaling	# 2947S
Cleaved caspase 3	Cell Signaling	# 9661S
HSP90	Cell Signaling	#4874S
NOS1	Fisher Scientific	PA1-033
NOS2	Fisher Scientific	PA3-030A
NOS3	Fisher Scientific	PA3-031A
Anti-rabbit IgG HRP-linked	Cell Signaling	#7074
Anti-mouse IgG HRP-linked	Cell Signaling	#7076
IRDye 800CW conjugated anti-rabbit	Li-COR Biosciences	#926-32211
IRDye 800CW conjugated anti-mouse	Li-COR Biosciences	#926-32210
AlexaFluor 680 conjugated anti-rabbit	Life Technologies	#A21109
AlexaFluor 680 conjugated anti-mouse	Life Technologies	#A21058
**Chemicals, peptides, and recombinant proteins**		
DMEM	Life Technologies	11965-084
Pen/Strep	Life Technologies	15140-122
HK-2 Keratinocyte SFM Media	Life Technologies	10724-011
SILAC Flex DMEM	Life Technologies	A24939-01
DMEM for Silac	Life Technologies	88364
Glucose-free DMEM	Life Technologies	11966-025
Glutamine	Life Technologies	25030-081
Standard FBS	Gemini	900-108
U-^13^C_6_-glucose	Cambridge Isotopes	CLM-1396
^15^N_2_-glutamine	Cambridge Isotopes	NLM-1328
^14^C-arginine	Perkin Elmer	NEC267E050UC
Glucose	Sigma Aldrich	G8270
Aspartate	Sigma Aldrich	A7219
Phenol red	Sigma Aldrich	P0290
WST-1	Sigma Aldrich	5015944001
Adenosine	Sigma Aldrich	A4036
Guanosine	Sigma Aldrich	G6264
Thymidine	Sigma Aldrich	T1895
Cytidine	Sigma Aldrich	C4654
Trolox	Cayman Chemical	10011659
*N*-acetyl-l-cysteine	Sigma Aldrich	A7250
Enhanced chemiluminescent substrate	Perkin Elmer	NEL105001EA
Matrigel matrix	Corning	354234
BioServ Dox Diet (200 mg/kg)	Fisher Scientific	S3888
Kidney clear cell carcinoma tissue array	US Biomax	OD-CT-UrKid02-003
Kidney clear cell carcinoma with matched kidney tissue array	US Biomax	KD601a
**Critical commercial assays**		
RNeasy Mini kit	Qiagen	#74104
High-Capacity RNA-to-cDNA kit	Applied Biosystems	#4368814
QuikChange II mutagenesis kit	Agilent	#200521
Click-iT™ Plus EdU Alexa Fluor™ 488 Flow Cytometry Assay Kit	Life Technologies	C10633
Proteome Profiler Human Angiogenesis Array Kit	R & D Systems	ARY007
NOS Activity Assay Kit	Cayman Chemicals	781001
**Experimental models: cell lines**		
HK-2	ATCC	CRL-2190
769-P	ATCC	CRL-1933
786-0	ATCC	CRL-1932
A498	ATCC	HTB-44
**Experimental models: organisms/strains**		
Mouse: NIH III nude, female homozygous	Charles River	#201
**Oligonucleotides**		
18S	Life Technologies	HS03928985_G1
ASS1	Life Technologies	HS01597989_G1
ASL	Life Technologies	Hs00902699_M1
**Recombinant DNA**		
pLKO.1 Scramble	Addgene	17920
LentiCRISPR v2	Addgene	52961
ASL ShRNA	GE Dharmacon	RHS3979-201794747
pCDH-CMV-MCS-EF1-Puromycin	System Biosciences	CD510B-1
ASS1 cDNA	System Biosciences	MHS1010-202694229
ASL cDNA	System Biosciences	MHS6278-202755499
**Software and algorithms**		
GraphPad Prism 8.0	GraphPad Software	https://www.graphpad.com/scientific-software/prism/
Spheroid macro	ImageJ	[30]

### Experimental model and subject details

#### Mice

The Institutional Animal Care and Use Committee (IACUC) at the University of Pennsylvania approved all mice xenograft experiments. Female NIH-III nude mice (Charles River, 6–8 weeks) were subcutaneously injected with 200 μL of 1:1 mixture of 5 million cells (in PBS) and Matrigel (Corning 356234). Tumor volume was monitored using caliper measurements. For doxycycline-induced expression of enzymes, xenograft injections were performed as above, and tumors were allowed to grow till 200 mm^3^ before switching the diet to a dox-containing chow (200 mg/kg). Mice were sacrificed using CO_2_ inhalation, and xenograft tumors were harvested for analyses.

#### Cell lines and cell culture conditions

ccRCC lines 769-P, 786-O, A498, RCC4, RCC10, UOK101, and UMRC2, along with immortalized renal epithelial line HK-2, were obtained from the American Type Culture Collection (ATCC). Cells were routinely tested for mycoplasma. HK-2 cells were cultured in serum-free keratinocyte medium supplemented with recombinant human epidermal growth factor and bovine pituitary extract, while ccRCC cell lines were grown in standard DMEM with 10% FBS and pen/strep. Hypoxia (0.5% O_2_) was used to study aspartic acid uptake in ccRCC cells. For labeled glucose and glutamine studies, cells were cultured with the labeled substrate for 24 h (10 mM U-^13^C_6_-d-glucose, low serum, 4 mM glutamine) and 3 h (4 mM ^15^N_2_ l-glutamine, low serum, 1 mM glucose).

### Method details

#### Viral transduction

HEK-292T cells were used in virus production. Cells were transfected with the plasmid of interest, pRSV-Rev, pMDL, and pCMV-VSV-G plasmids combined with Fugene6, and the virus was harvested after 48 h. Experimental lines were generated with 24-h viral transduction followed by treatment with an antibiotic selection marker.

The pLKO.1 lentiviral transduction system from Addgene was used with short hairpins against ASL (GE Dharmacon) to generate stable knockdowns. Similarly, pCDH-CMV-MCS-hygro/puro plasmids were utilized to express ASL cDNA in ccRCC cells. Additionally, the QuikChange II mutagenesis kit was used to generate mutant ASL clones.

#### Western blot analysis

HEPES containing buffer supplemented with 1% Triton-X and protease inhibitors was used for cell lysis. Xenograft tumors were homogenized in the lysis buffer using a tissue tearator prior to analysis. Samples were run on appropriate SDS containing gels following protein quantitation using BCA assay. Proteins were transferred to nitrocellulose membranes (Biorad #162-0115, 0.45 μm pore size) and incubated overnight at 4 °C with primary antibodies in TBST (20 mM Tris, 135 mM NaCl, and 0.02% Tween 20) with 5% BSA. Secondary antibodies conjugated with HRP were used, followed by visualization with ECL reagents.

#### TCGA RNA-seq analysis

RNA-seq expression data was downloaded from The Cancer Genome Atlas (TCGA) Clear Cell Renal Cell Carcinoma project, and metabolic gene sets were generated using the Kyoto Encyclopedia of Genes and Genomes (KEGG) (http://www.genome.jp/kegg/). Ranked set lists were calculated using log2 fold change between ccRCC tumors vs normal kidney tissues. Survival analysis was performed in Prism, and Kaplan-Meier curves were plotted with statistical effects.

#### Immunohistochemistry

Matched tumor-normal patient microarray slides (BioMax KD601, OD-CT-UrKid03-002) were rehydrated using xylenes and a series of ethanol solutions. Peroxidase activity was blocked by treatment with 1% H_2_O_2_, and antigen retrieval was carried out with a citrate unmasking solution. Slides were blocked in a goat serum containing buffer and incubated overnight with primary antibodies. The next day after TT buffer washes, slides were incubated with biotinylated secondary antibodies, and sequentially processed with an ABC kit and a DAB peroxidase substrate kit. Stained slides were dehydrated with ethanol series and xylenes and mounted using a permount solution.

#### qRT-PCR

RNA was extracted using the RNeasy kit, and cDNA was synthesized using the high-capacity RNA to cDNA kit. A ViiA7 Real-Time PCR machine was used for the qRT-PCR. Following TaqMan, primers were used—18S (HS03928985_G1), ASS1 (HS01597989_G1), and ASL (Hs00902699_M1).

#### Soft agar colony-forming assay

Cells were seeded at a density of 6000 cells/well in standard DMEM with 10% FBS and 0.3% agarose (low-melt 2-hydroxyethylagarose, Sigma Aldrich A4018) and overlaid onto a cell-free layer of DMEM and 0.6% agarose. Fresh agarose-containing media was added weekly, and colonies were counted at the end of three weeks.

#### Matrigel-based spheroids

The protocol was adapted from [37, 38]. A total of 3000 cells were seeded in each well of a 96-well low-adherence plate in DMEM supplemented with 10% FBS and 2.5% Matrigel, followed by low-speed centrifugation to promote spheroid formation. Images were captured using an EVOS FL Auto Imaging System, and spheroid volume was estimated using an ImageJ macro [39].

#### 2D cell growth assays

A total of 100,000 cells/well were plated in a 6-well plate in low-serum (1%) DMEM for each of the HK-2 cell lines, and cells were counted on days 0, 2, 4, and 6 using an automated cell counter (Countess). In other cases, WST-1 was used to calculate the cell numbers based on relative absorbance. The manufacturer’s protocol was followed, the media were changed every alternate day, and all readouts were normalized to day 0 values.

#### Steady-state metabolomics

One million cells were plated in a 15-cm tissue culture dish and allowed to acclimatize for 24 h in complete DMEM before switching to 1% FBS containing DMEM. After 48 h, the media were aspirated, and cells were washed with ice-cold PBS. In a cold room, liquid nitrogen was poured directly onto the dish and allowed to boil over and evaporate. The resulting frozen monolayer of cells was scraped into a 50-mL conical tube and thawed, and an aliquot was set aside for protein measurement. Aliquots (100 μL) of thawed cell lysates on ice were homogenized in equal volumes of acetonitrile/0.6% formic acid followed by vortexing for several seconds to lyse cells. Amino acids and their isotopically labeled internal standards were extracted from 100 μL aliquots of cell homogenates using 800 μL of ice-cold methanol. This mixture was vortexed for several seconds followed by centrifuging at 14000×*g* for 10 min at 4 °C. A 100-μL aliquot of each methanol supernatant was dried under nitrogen, and amino acids were derivatized with a quinoline functional group. Derivatized amino acids and their isotopically labeled internal standards were quantitated using an Agilent 1290 Infinity UHPLC/6495B triple quadrupole mass spectrometer. Multiple reaction monitoring was used to quantitate a fragment ion of the parent ion of each amino acid with standard calibration curves. Concentrations of amino acids were normalized to the protein concentration of each cell lysate.

#### Labeled metabolite tracing

For labeled glucose and glutamine studies, cells were cultured with the labeled substrate for 24 h (10 mM U-^13^C_6_-d-glucose, low serum, 4 mM glutamine) and 3 h (4 mM ^15^N_2_ l-glutamine, low serum, 1 mM glucose), and then processed for isotope measurement. The protocol was adapted from [40, 41]. Cells were washed twice with ice-cold 1× PBS and then scraped on ice in 4% perchloric acid (PCA). This was followed by three freeze-thaw cycles. Thawed cell extracts were neutralized with 5 M KOH, centrifuged, and passed through an AG-1 column (Biorad), for separation of organic acids, glutamate, and aspartate, before further derivatization with t-butyldimethylsilyl (TBDMS). For [^13^C] aspartate, m/z ratios at 418, 419, 420, 421, and 422 for M0, M1, M2, M3, and M4 (containing 1 to 4 ^13^C atoms above M0, the natural abundance), respectively, were monitored and measured using GC-MS. Similarly, for measurement of ^15^N enrichment in aspartate, m/z ratios between 419/418 were monitored following TBDMS derivatization. For ^13^C enrichment in orotic acid (OA), m/z ratios at 441,442,443, 444, 445, and 446, ^13^C enrichment in ASA was measured using the butylation derivatization method. The sample was dried down, then 200 μL of 3N HCL in butanol was added. Heated for 15 min at 60 °C, and then, cooling down the sample and reconstituted in 100 μL of solution A (0.1% formate in water). Measurements were performed using LC-MS (Agilent 1260 LC combined with triple-quad 6410B Mass Spectrometer), with LC gradient of solution A and solution B (acetonitrile with 0.1% formate and 0.005% TFA). Separation was performed with Poroshell 120 EC-C15 column. The ^13^C enrichment in M1 to M6 of ASA was determined using MRM 459-214 to 465-214 in positive mode.

#### NO metabolite measurement

Homogenates were prepared by lysing tissues in a HEPES-containing buffer using a tissue tearator, and a small aliquot was set aside for protein quantitation. All samples were filtered through a 10-kDa cutoff filter prior to analysis. Nitric oxide metabolites were quantified using a Sievers nitric oxide analyzer (Sievers Instruments, Boulder CO), as previously described by J.O. Lundberg, and M. Govoni [42]. Briefly, samples were injected into a reaction chamber containing vanadium (III)/hydrochloric acid solution heated to 95 °C. The NO generated from the reduction of nitrate, nitrite, and *S*-nitrosothiols was quantified via a reaction with ozone by gas-phase chemiluminescence. Known concentrations of nitrate ranging from 100 to 1.6 μM were injected, and a standard curve was generated. Signal peaks (mV) were manually integrated, and the areas were used for quantification of NO metabolite concentration.

#### NOS activity assay kit

Tissues were homogenized in the provided homogenization buffer and centrifuged, and the supernatant was collected and kept on ice till further processing. The reaction mixture was prepared with the provided reaction buffer, 10 mM NADPH, CaCl_2_, and ^14^C arginine (100 μCi/mL). The tissue supernatants were incubated with the reaction buffer for 60 min at room temperature. The equilibrated resin was added to the reaction sample and moved to the spin columns provided. The samples were spun down, and the follow through was collected and processed on a scintillation counter. Appropriate controls were run, and percent citrulline was estimated from total counts.

#### Cell cycle analysis

Cells were plated at 60% confluency and treated with 20 μM lovastatin for 24 h (G1 block), a double thymidine block (2 mM thymidine for 12 h followed by an 8-h treatment with 25 μM deoxycytidine, and 2 mM thymidine again for 12 h, S phase block). RO3306 and nocodazole treatment was used post the double thymidine block for G2 and mitotic cells, respectively. Harvested cells were centrifuged, resuspended in PBS, fixed overnight with 100% ethanol, and stained with propidium iodide (PI) for 20 min. BD FACS Calibur and FlowJo were used for the experiment and analyses.

#### Click-it EdU flow cytometry assay

Cells were incubated with EdU (5-ethynyl-2′-deoxyuridine) for 24 h and harvested following the kit protocol. Briefly, cells were trypsinized, spun down, fixed with 4% paraformaldehyde, washed with PBS, and incubated with the dye in a saponin-based buffer for 30 min in the dark. This was followed by flow cytometry on BD FACS Calibur and analyses on FlowJo.

#### Proteome profiler human angiogenesis array

Xenograft tumors were lysed in PBS containing protease inhibitors and processed according to the kit protocol. The lysates were incubated with a series of buffered protein base solutions on a rocking platform, before being incubated with the antibody cocktail and left overnight. The next day, the membranes were washed and incubated with HRP-Streptavidin containing buffer, and X-ray exposure was carried out using chemiluminescent agents. The ImageJ software was used to analyze the intensities of the dot blot generated from the array, and a heatmap was plotted using GraphPad Prism.

#### Quantification and statistical analysis

GraphPad Prism was used for all statistical analyses. Error bars represent mean ± *S.E.M.* Figure legends indicate the pertinent tests used along with *p*-values.

## Results

### Urea cycle enzymes ASS1 and ASL exhibit diminished expression in ccRCC tumors

We performed metabolic gene set analysis on mRNA expression data from The Cancer Genome Atlas (TCGA) now including 538 ccRCC tumors and 72 normal kidney samples [8, 43]. As shown previously [18], we ascertained that the genes encoding enzymes of the urea cycle are underexpressed in ccRCC tumors when compared to normal kidneys (Fig. 1A, B). As the importance of repressing mitochondrial ARG2 as a metabolic tumor suppressor in ccRCC [18] has been established, we now focused on the cytosolic urea cycle enzymes ASS1 and ASL that convert citrulline and aspartate to arginine and fumarate via argininosuccinate. Survival data from TCGA indicated that patients with high combined expression of *ASS1* and *ASL* (*n* = 92) exhibited significantly improved survival rates compared to those with low expression of both transcripts (*n* = 352) (Fig. 1C and Supp. Figure 1 A-B), while single genes on their own did not significantly alter survival. Furthermore, both *ASS1* and *ASL* mRNAs are consistently underexpressed in ccRCC tumors when compared to normal kidney tissues (Fig. 1D), especially *ASS1*. This is also reflected in the lowered protein abundances of ASS1 and ASL in 50 matched primary patient tumor-normal cores (Fig. 1E and Supp Figure 1 C). Finally, most ccRCC cell lines have decreased ASS1 and ASL when compared to the immortalized renal epithelial cell line, HK-2 (Fig. 1F), with the exception of 769-P and UMRC2, providing a means of assessing their impact upon re-expression.

### Altered ASL expression impacts growth in normal kidney and ccRCC cells

**Fig. 2:**
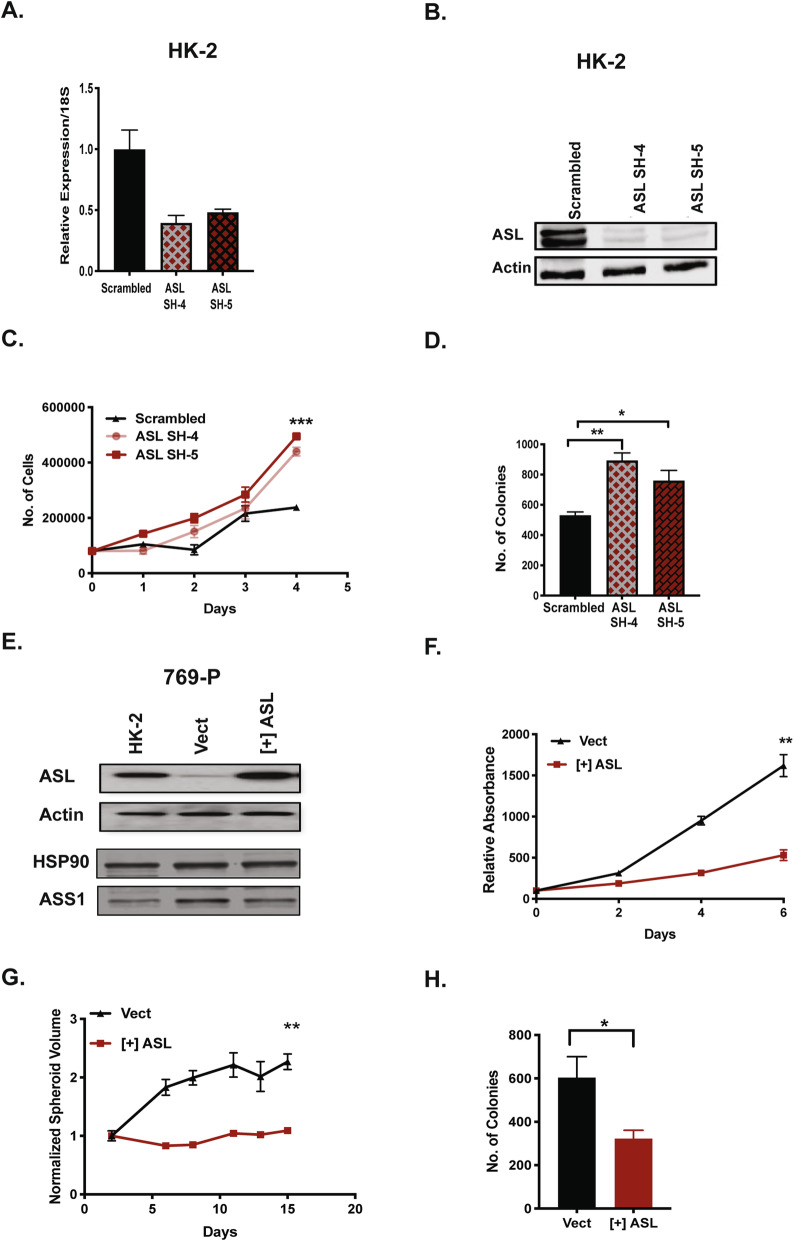
ASL expression alters growth in normal kidney and ccRCC cells. **A** mRNA expression levels of *ASL* in HK-2 cells post-knockdown with two independent shRNAs. Scrambled shRNA was used as a control. **B** Protein levels of ASL in HK-2 cells after shRNA knockdown. Actin is the loading control. **C**, **D** ASL knockdown enhances HK-2 cell growth in **C** 2D and **D** 3D soft agar colony-forming assay. In **C**, cells were grown in 1% FBS and standard DMEM. Error bars represent *SEM* from three replicate wells. **p < 0.01, *p < 0.05. **E** Ectopic expression of ASL in 769-P ccRCC polyclonal populations. HK-2 cells were used as physiologic control. HSP90 and Actin serve as loading controls. **F**–**H** ASL re-expression in 769-P cells suppresses growth in **F** 2D, **G** 3D matrigel spheroids, and **H** 3D soft agar colony-forming assay. 769-P cells expressing empty vector were used as a control. In **F**, cells were grown in 1% FBS and standard DMEM. **p < 0.01, *p < 0.05

In order to recapitulate ASL loss in tumor cells, we employed shRNA-mediated knockdown of ASL in normal HK-2 renal epithelial cells (Fig. 2A, B) with two independent constructs (SH-4, SH-5), while a scrambled shRNA was used as control. ASL loss enhanced growth in HK-2 cells in 2D culture (Fig. 2C) and a 3D soft agar colony formation assay (Fig. 2D). Re-expression of ASL alone in 786-O and UMRC2 cells modestly suppressed growth in a 3D soft agar colony-forming assay (Supp Figure 2). Similar results were obtained for ASS1 re-expression in ccRCC [18]. With the aim of combined restoration of both ASS1 and ASL, we introduced a vector encoding ASL in 769-P cells that have substantial remaining ASS1 expression (Fig. 2E). This in turn significantly suppressed growth in 2D culture (Fig. 2F), where cells were grown in low-serum (1% FBS) conditions. Furthermore, this growth reduction was reproduced in a 3D Matrigel-based spheroid assay and soft agar colony formation assay (Fig. 2G, H). We concluded that like ARG2 and ASS1, ASL can serve as a metabolic tumor suppressor in ccRCC.

### Combined re-expression of ASS1 and ASL suppresses growth and alters aspartate metabolism in ccRCC cells in a catalytically dependent manner

**Fig. 3:**
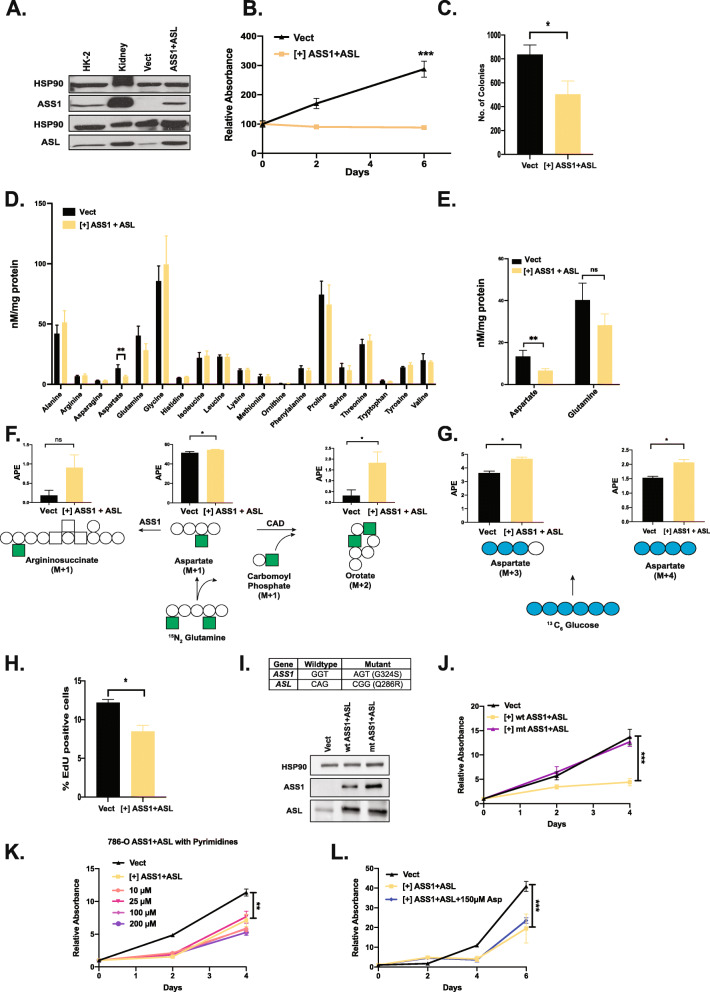
Combined expression of ASS1 and ASL in ccRCC cells suppresses growth and alters the metabolic landscape in a catalytically dependent manner. **A** Combined ectopic expression of ASS1 and ASL in 786-O polyclonal population. HSP90 serves as the loading control, while HK-2 cells and the normal kidney are normal controls. **B** ASS1+ ASL expression suppresses growth in 786-O cells in a 2D growth assay. Error bars represent *SEM* of 7 wells, and cells were grown in 1% FBS in complete DMEM. ***p < 0.001. **C** 3D soft agar colony-forming assay with 786-O cells expressing empty vector control and ASS1+ASL cDNA. Error bars represent *SEM* of 3 wells. *p < 0.05. **D** Steady-state amino acid levels in 786-O cells expressing empty vector and ASS1+ASL cDNA. Error bars represent SEM of 3 technical replicates. **p < 0.01. **E** Steady-state aspartate and glutamate levels in 786-O cells expressing empty vector and ASS1+ASL cDNA. Error bars represent *SEM* of 3 technical replicates. **p < 0.01. **F** Schematic representation of ^15^N_2_-glutamine labeling into aspartate, orotate, and argininosuccinate. Argininosuccinate synthase (ASS1), carbamoyl-phosphate synthetase 2, aspartate transcarbamylase, and dihydroorotase (CAD enzyme complex). The filled-in squares represent heavy nitrogen (^15^N) while clear circles represent carbon atoms (^12^C). **G** Schematic representation of ^13^C_6_-glucose labeling into aspartate. The filled-in circles represent heavy carbon atoms (^13^C). **H** EdU incorporation assay with 786-O cells expressing empty vector and ASS1+ASL cDNA. Error bars represent *SEM* of 3 technical replicates. *p < 0.05. **I** Combined ectopic expression of wild-type and catalytically dead mutant ASS1 and ASL in 786-O polyclonal population with mutant sequence. **J** ASS1+ASL-mediated growth suppression in 786-O cells is dependent on their catalytic activity as seen in a 2D growth assay. Error bars represent *SEM* of 6 technical replicates. ***p < 0.001. **K** 786-O cells expressing ASS1+ ASL cDNA were cultured with exogenously provided pyrimidines (cytidine, thymidine, and uridine) at indicated concentrations. 786-O cells expressing empty vector are shown as control. Error bars represent *SEM* of 6 technical replicates. **p < 0.01. **L** 786-O cells expressing ASS1+ASL cDNA were cultured with 150 μM exogenously provided aspartic acid. 786-O cells expressing empty vector are shown as control. Error bars represent *SEM* of 6 technical replicates. ***p < 0.001

The 769-P ccRCC cell line has several limitations, chief among them is their inability to form in vivo xenograft tumors. In order to explore ASS1- and ASL-mediated growth suppression in this setting, we re-expressed ASS1 and ASL together in 786-O cells at levels approximating HK-2 cells (Fig. 3A). Of note, ASL levels in transduced 786-O cells were comparable to human kidney tissue, while ASS1 levels were lower, reflecting the intrinsic growth suppression of ASS1 restoration. Nevertheless, ASL/ASS1 re-expression significantly reduced 2D growth (1% FBS) (Fig. 3B) and soft agar colony formation (Fig. 3C). To further elucidate the effects of combined ASS1 and ASL re-expression in 786-O cells, we measured steady-state levels of intracellular amino acids by mass spectrometry (Fig. 3D). Combined ASS1 and ASL altered amino acid levels somewhat, especially cellular aspartate, which decreased significantly. Aspartate is vital to cellular growth, and lowered aspartate levels can have detrimental effects on pyrimidine production among other things [20, 23, 24]. Cells attempt to make up for deficits in intracellular aspartate by generating more from glucose and glutamine (Fig. 3E–G). This imposes an additional metabolic burden on them and slows proliferation, specifically DNA production as evidenced by an EdU incorporation assay (Fig. 3H). We re-expressed catalytically dead versions of ASS1 and ASL in 786-O cells (Fig. 3I), which were identified from the literature and reported to have < 2% of enzymatic activity [44, 45]. The catalytically dead mutants failed to suppress growth in 786-O cells (Fig. 3), thus indicating that ASS1- and ASL-mediated suppression is contingent on their enzymatic activity. ASL re-expression in 769-P cells also showed similar trends in intracellular aspartate and glutamine levels (Supp. Figure 3 A). Additionally, 769-P cells expressing ASL exhibit a peculiar sub-G0 peak (Supp Figure 3 B) in cell cycle experiments hinting at aberrant pyrimidine synthesis and DNA replication. The addition of exogenous pyrimidines to the culture medium rescued growth in 769-P cells (Supp. Figure 3 C). However, we were unable to rescue growth in 786-O cells with exogenously provided pyrimidines or aspartate (Fig. 3K, L). Recent studies have shown that upregulation of antioxidants and nucleotide production can transform cells [46]; however, the addition of antioxidants Trolox (100uM) or *N*-acetylcysteine (NAC, 1 mM) alone or in combination with 50 μM nucleosides did not rescue the proliferation defects of 786-O cells expressing ASS1 and ASL (Supp Figure 3 D). This points towards the involvement of additional factors in ASS1+ASL-mediated suppression.

### Combined re-expression of ASS1 and ASL suppresses ccRCC growth in vivo

**Fig. 4:**
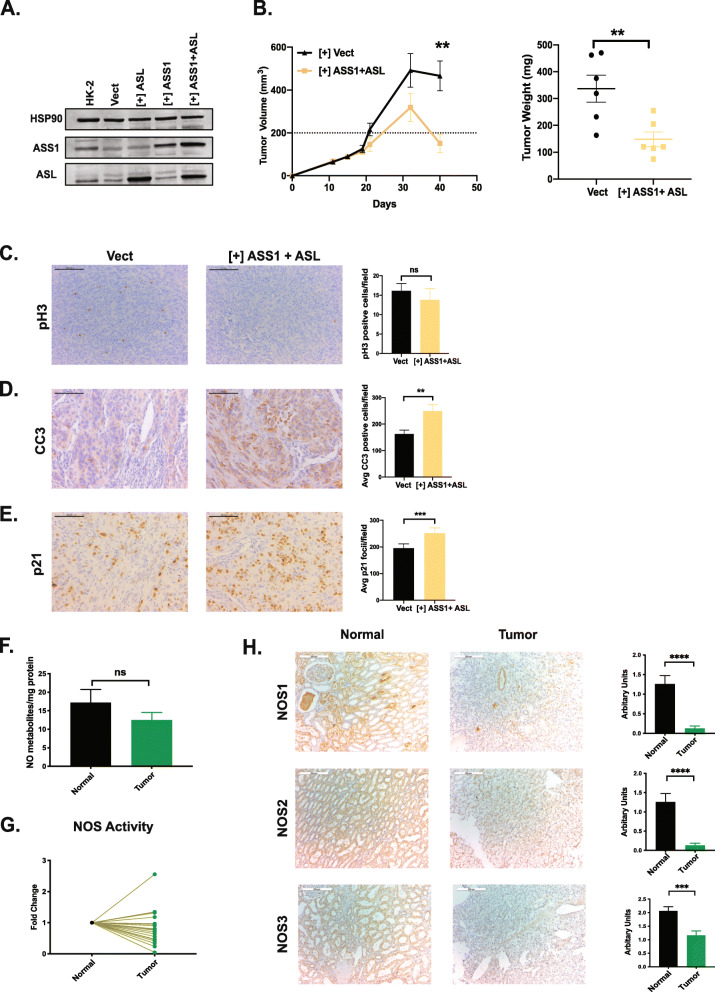
Combined re-expression of ASS1 and ASL suppresses growth in vivo. **A** Combined doxycycline-induced ectopic expression of ASS1+ ASL in 786-O cells. HSP90 is used as a loading control. **B** 786-O subcutaneous xenograft tumor growth in nude mice, where cells have doxycycline induced expression of ASS1 and ASL or empty vector (*n* = 6). **C** Immunohistochemistry staining and quantification for phosphorylated histone H3 as a marker for proliferation in xenograft tumors. Scale bars are at 100 μm. **D** Immunohistochemistry staining and quantification for cleaved caspase 3 as a marker for apoptosis in xenograft tumors. Scale bars are at 100 μm. **p < 0.01. **E** Immunohistochemistry staining and quantification for p21 as a marker for proliferation in xenograft tumors. Scale bars are at 100 μm. ***p < 0.001. **F** Nitric oxide metabolite levels in matched ccRCC tumors and normal kidney as measured using gas-phase chemiluminescence (*n* = 20). **G** Nitric oxide synthase activity measured by radiolabeled citrulline generation, plotted as a fold change between matched normal and ccRCC tumor tissues (*n* = 20). **H** Representative immunohistochemistry images with quantification for NOS1,2,3 protein expression in matched tumor-normal pairs (Biomax OD-CT-UrKid03-002) (*n* = 31). Scale bars represent 200 μm. ****p < 0.001, paired *t*-test

There is a strong selective pressure against ccRCC cells with combined ASS1/ASL re-expression and maintaining physiological levels has been inconsistent (data not shown). To overcome this, we employed a doxycycline-inducible system to allow acute restoration of these enzymes (Fig. 4A). Sustained, combined ASS1/ASL re-expression under doxycycline control significantly suppressed growth in a subcutaneous 786-O xenograft model, where mice were fed chow supplemented with doxycycline (200 mg/kg) (Fig. 4B). These xenografts exhibited lowered phosphorylated histone H3 (pH 3) levels which did not achieve statistical significance (Fig. 4C), enhanced cleaved caspase 3 (CC3) (Fig. 4D), and increased p21 based on immunohistochemistry (Fig. 4E). These analyses indicate slower proliferation and increased apoptosis and defects in the cell cycle. It is conceivable that aspartate availability is further limited in a harsher in vivo setting, augmenting the growth suppression observed in vitro. Interestingly, 786-O cells with ASL re-expression alone (Supp. Figure 3 A) exhibit modest growth suppression in vivo (Supp. Figure 4 A) limited to tumor volumes and weights. Moreover, immunohistochemical analyses for pH3, CC3, and p21 were not significantly different (Supp. Figure 4 B-D). Of note, 786-O xenograft tumors with only ASL re-expression displayed significantly decreased levels of CD31 (Supp Figure 4 E), an endothelial cell marker, indicating abnormalities in tumor vasculature. We assessed levels of various angiogenic proteins in these tumors using a membrane-based sandwich immunoassay (Supp Figure 4 F). ASL restored 786-O xenograft tumors broadly altered the levels of angiogenic proteins (e.g., VEGF, collagen XVIII, TIMP-1). The exact mechanism of these changes is unknown but can be associated with nitric oxide synthesis. Due to the lack of a sensitive assay to measure intracellular nitric oxide (NO) levels, we resorted to the measurement of downstream NO metabolites in matched tumor-normal pairs. Human ccRCC patient tumors exhibited lower levels of NO metabolites (Fig. 4F) when compared to matched normal kidney tissue. Furthermore, we employed radiolabeled arginine to assess nitric oxide synthase (NOS) activity in ccRCC tumors and matched normal patient samples, which we found to be lowered in the tumors (Fig. 4G). This is in line with previously published data [47]. We also observed lowered NOS activity in ccRCC cell lines when compared to normal HK-2 cells (data not shown). Furthermore, we observed lowered expression of NOS isoforms (NOS1, 2, and 3) in ccRCC patient tumors when compared to matched normal tissue (Fig. 4H). Lowering of NOS activity and subsequent NO generation might aid cell proliferation as NO is known to trigger apoptosis [48]. Thus, in addition to aspartate conservation, ccRCC tumors likely reduce ASS1 and ASL expression to curb NO generation and circumvent its cell-intrinsic anti-proliferative effects.

## Discussion

Despite strides made in the development of VEGF and HIF2-α antagonists [49], ccRCC patients still lack a sufficient number of therapeutically relevant genetic and signaling targets. The altered metabolic landscape of these tumors offers unique vulnerabilities that warrant exploration [5]. Our study here uncovers two potential metabolic tumor suppressors in ccRCC—the urea cycle enzymes ASS1 and ASL.

Collating data from TCGA, matched patient samples, and ccRCC cell lines, we establish a widespread loss of expression of urea cycle genes *ASS1* and *ASL*, along with a significant reduction in their protein levels. Furthermore, we demonstrate that their expression in ccRCC patients dictates survival. This is in addition to the near universal loss of the urea cycle enzyme ARG2 and lowered abundances of urea cycle intermediates in ccRCC tumors [18].

Although the loss of ASS1 expression is reported in a variety of cancers [20], we show reduced expression of both ASS1 and ASL in ccRCC tumors. We model ASL loss in HK-2 cells using short hairpin RNAs and observe increased proliferation. Furthermore, either ASS1 and ASL re-expression in ccRCC cells produces modest growth suppression, while their combined expression significantly reduces growth in 2D, 3D, and subcutaneous xenograft models. ASS1 and ASL have previously been described as potent regulators of cell growth, and our data establishes their role in the ccRCC milieu.

To further explore the metabolic consequences of ASS1 and ASL re-expression, we performed metabolite measurements at steady state and by tracing U-^13^C_6_-d-glucose and ^15^N_2_ l-glutamine. We observed decreased steady-state aspartate and glutamate abundance. Interestingly, we also detected increased label enrichment in aspartate from glucose and glutamine. As reported earlier, aspartate is a key metabolite crucial for nucleotide synthesis, proteogenesis, and TCA anaplerosis in rapidly dividing cells [20, 23, 24]. ASS1 and ASL re-expression shunts cellular aspartate pools towards ureagenesis, forcing the cell to enhance synthesis to keep up with demand. This levies an additional metabolic pressure on these cells and contributes to growth retardation.

In addition to being part of the urea cycle, the reactions catalyzed by ASS1 and ASL also contribute to the citrulline-nitric oxide (NO) cycle [50]. We observed reduced levels of NO metabolites and nitric oxide synthase expression in ccRCC tumors and cell lines. NO is a potent regulator of numerous cellular processes including growth and angiogenesis [48]. Alterations in NO metabolism via reduced substrate availability or enzymatic activity could help explain the cell non-autonomous effects of ASS1 and ASL loss in ccRCC.

We have previously shown that ARG2 loss conserves pyridoxal phosphate (PLP) in ccRCC cells and tumors [18]. Interestingly, PLP is also required for cellular aspartate synthesis. Therefore, combined ASS1, ASL, and ARG2 downregulation together contributes towards the overall conservation of intracellular aspartate—indirectly by conserving PLP and directly by diverting aspartate away from the urea cycle. It is interesting to note that all three enzymes suppress ccRCC growth in a catalytically dependent fashion as re-expression of enzyme dead mutants failed to alter the growth kinetics in ccRCC cells. Previous studies have pointed towards the formation of a tripartite complex consisting of ASS1, ASL, and NOS necessary for NO generation in endothelial cells where ASS1 and ASL serve as adaptors [50] However, the complete reliance of ASS1 and ASL on their enzymatic activity to suppress ccRCC growth precludes any catalytic activity-independent contributions in this context.

## Conclusions

Our data indicate that the nearly universal loss of ASS1 and ASL in ccRCC tumors promotes growth by conserving intracellular aspartate pools, ostensibly for pyrimidine synthesis, and by regulating nitric oxide synthesis to provide cells a proliferative advantage. We also ascertained the catalytic dependence of this phenotype, as expression of enzymatically dead versions of ASS1 and ASL abrogates the growth suppression. Furthermore, we observed decreased nitric oxide (NO) production in ccRCC tumors and cells lacking ASS1 and ASL. We hypothesize that loss of ASS1 and ASL alters cellular NO metabolism and aids cell proliferation by regulating the cytotoxic effects of NO generation.

In conclusion, our data establishes context-specific effects of urea cycle enzymes ASS1 and ASL, while highlighting the novel metabolic vulnerabilities in ccRCC tumors.

## Supplementary Information

**Additional file 1: Figure S1.** *ASS1* and *ASL* expression affects survival in ccRCC patients. A. Survival curve based on *ASS1* expression in patients from the TCGA. (High *n*=176, low *n*=352). B. Survival curve based on *ASL* expression in patients from the TCGA (High *n*=258, low *n*=270). C. Log2 scale fold change in ASS1 and ASL protein levels in tumors when compared to normal kidney (*n*=108, Clark *et al*.).

**Additional file 2: Figure S2.** ASL Expression Suppresses Growth in a ccRCC cells. A. Ectopic expression of ASL in 786-O polyclonal population. Actin is used as the loading control. B. 3D soft agar colony forming assay with 786-O cells expressing empty vector control and ASL cDNA. **p< 0.01. C. Ectopic expression of ASL in UMRC2 polyclonal population. Actin is used as the loading control. D. 3D soft agar colony forming assay with UMRC2 cells expressing empty vector control and ASL cDNA. **p< 0.01.

**Additional file 3: Figure S3**. ASL Expression in 769-P cells Alters Amino Acid Pools and Affects DNA Replication. A. Steady-state amino acid levels in 769-P cells expressing empty vector and ASL cDNA. Error bars represent SEM of 3 technical replicates. ****p< 0.001, ***p< 0.001. B. Representative images of cell cycle analyses of 769-P cells with propidium iodide (PI) staining indicating a sub-G0 peak in cells expressing ASL cells at 0 hours and 24 hours. C. Exogenously provided pyrimidines (cytidine + thymidine, 50 μM) partially rescue growth in 769-P cells expressing ASL. Error bars represent SEM of 7 wells. *p< 0.05, ***p< 0.001. D. 786-O cells expressing ASS1+ASL cDNA were cultured with Trolox (Trol, 100μM), N-Acetylcysteine (NAC, 1mM), and/or nucleosides (adenosine + guanosine + cytidine + thymidine, 50μM) and assessed for cell proliferation by WST-1. Error bars represent SEM of 7wells. ***p< 0.001.

**Additional file 4: Figure S4.** Re-expression of ASL Alone in 786-O Cells Does Not Suppress Growth in vivo. A. 786-O subcutaneous xenograft tumor growth in nude mice, where cells expression ASL or empty vector. (*n* = 5). B. Immunohistochemistry staining and quantification for phosphorylated histone H3 as marker for proliferation in xenograft tumors. Scale bars are at 100μm. C. Immunohistochemistry staining and quantification for cleaved caspase 3 as marker for apoptosis in xenograft tumors. Scale bars are at 100μm. D. Immunohistochemistry staining and quantification for p21 as marker for proliferation in xenograft tumors. Scale bars are at 100μm. E. Immunohistochemistry staining and quantification for CD31 as marker for vasculature in xenograft tumors. Scale bars are at 100μm. *p< 0.05. F. Heat map showing the levels of various angiogenic proteins in 786-O xenograft tumors expressing ASL or empty vector.

## Data Availability

This manuscript includes all data generated and analyzed in this study. More information is available from the corresponding author on request.

## Abbreviations

ASL: Argininosuccinate lyase
ASS1: Argininosuccinate synthase 1
CC3: Cleaved caspase 3
ccRCC: Clear cell renal cell carcinoma
DMEM: Dulbecco’s modified Eagle’s medium
EdU: 5-Ethynyl-2′-deoxyuridine
FBS: Fetal bovine serum
NO: Nitric oxide
NOS: Nitric oxide synthase
OA: Orotic acid
pH 3: Phosphorylated histone 3
PI: Propidium iodide
PLP: Pyridoxal-5-phosphate
shRNA: Short hairpin RNA
TCGA: The Cancer Genome Atlas
