# Supplementary figures and images for: ASS1 and ASL suppress growth in clear cell renal cell carcinoma via altered nitrogen metabolism
Source: Cancer Metab. 2021 Dec 3;9:40. doi: 10.1186/s40170-021-00271-8 (PMC8642968; doi:10.1186/s40170-021-00271-8)

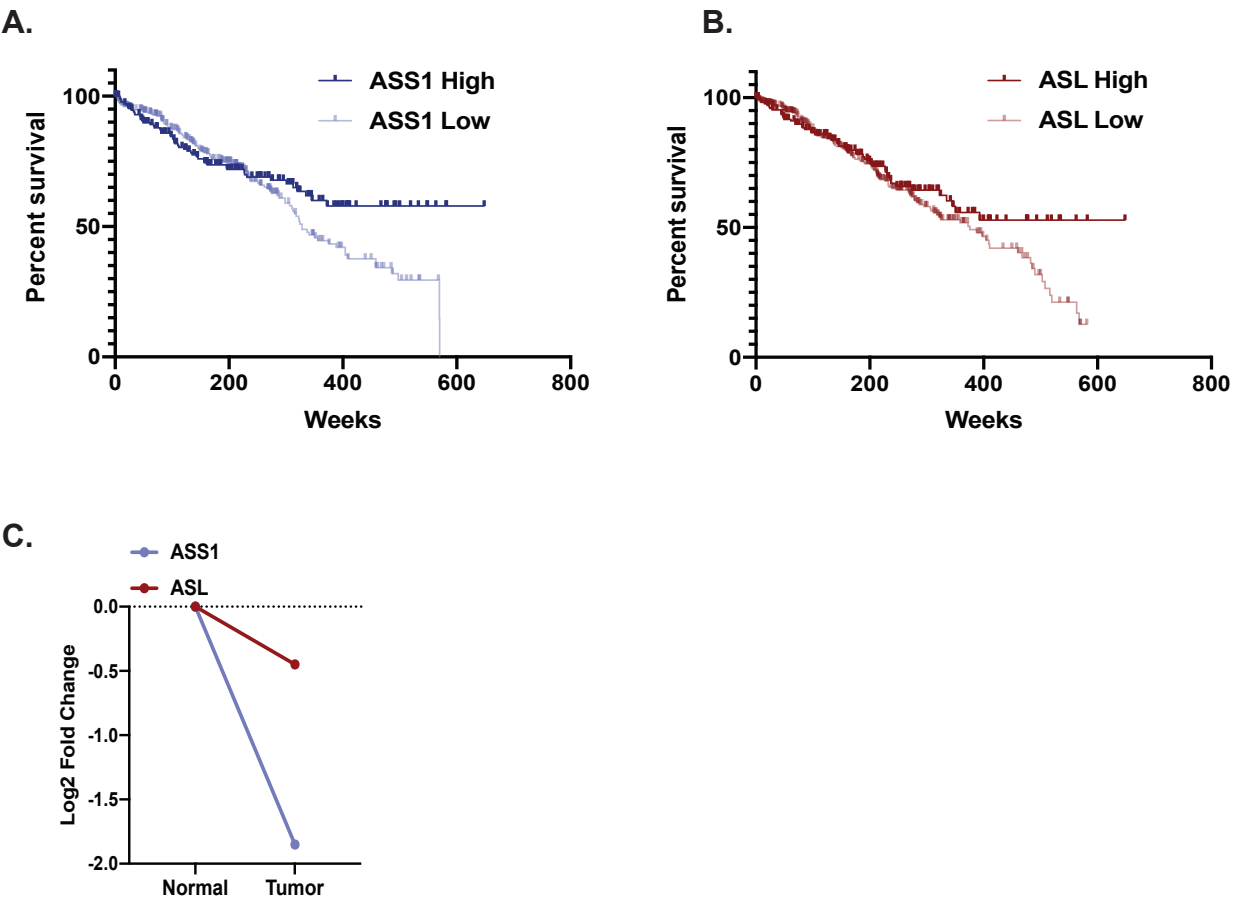

Supp. Figure 1

Supplement: Supplementary file 1 — Additional file 1: Figure S1. ASS1 and ASL expression affects survival in ccRCC patients. A. Survival curve based on ASS1 expression in patients from the TCGA. (High n=176, low n=352). B. Survival curve based on ASL expression in patients from the TCGA (High n=258, low n=270). C. Log2 scale fold change in ASS1 and ASL protein levels in tumors when compared to normal kidney (n=108, Clark et al.). [file 40170_2021_271_MOESM1_ESM.pdf]

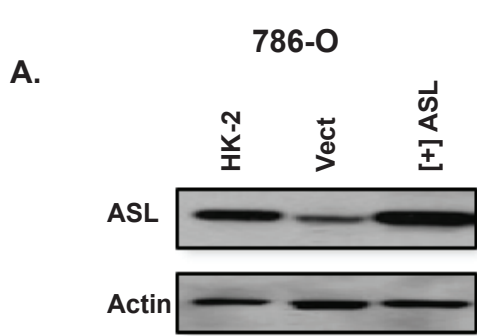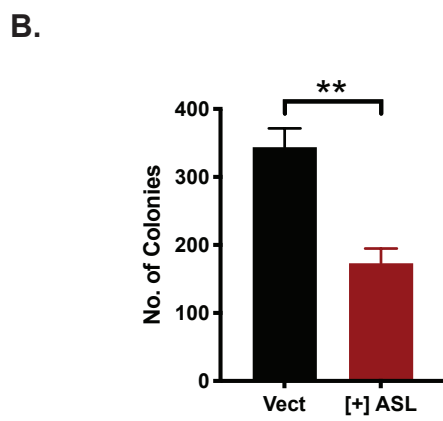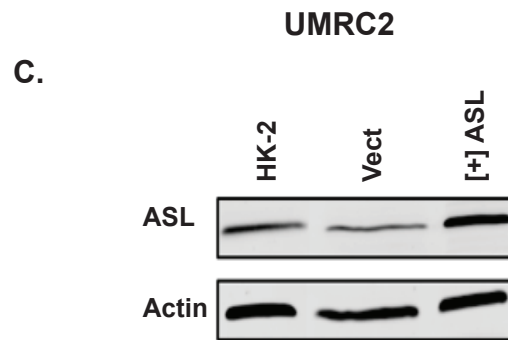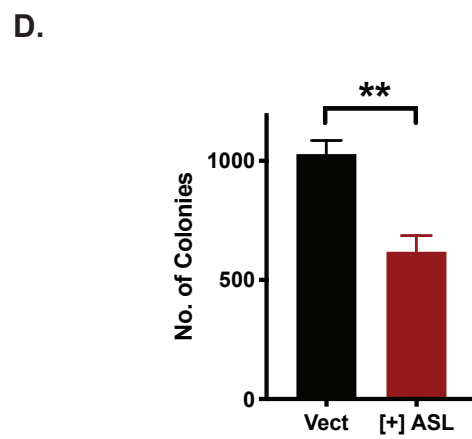

Supp. Figure 2

Supplement: Supplementary file 2 — Additional file 2: Figure S2. ASL Expression Suppresses Growth in a ccRCC cells. A. Ectopic expression of ASL in 786-O polyclonal population. Actin is used as the loading control. B. 3D soft agar colony forming assay with 786-O cells expressing empty vector control and ASL cDNA. **p< 0.01. C. Ectopic expression of ASL in UMRC2 polyclonal population. Actin is used as the loading control. D. 3D soft agar colony forming assay with UMRC2 cells expressing empty vector control and ASL cDNA. **p< 0.01. [file 40170_2021_271_MOESM2_ESM.pdf]

A.

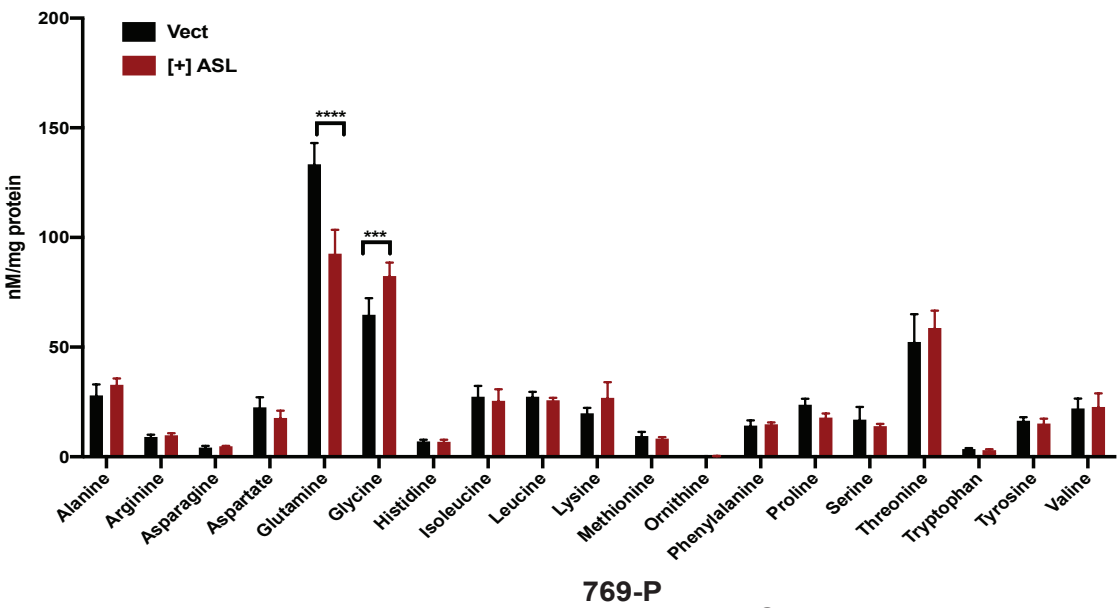

B.

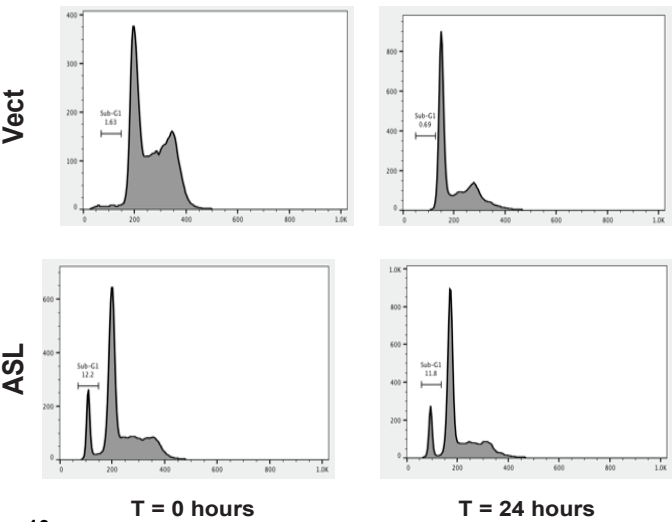

C.

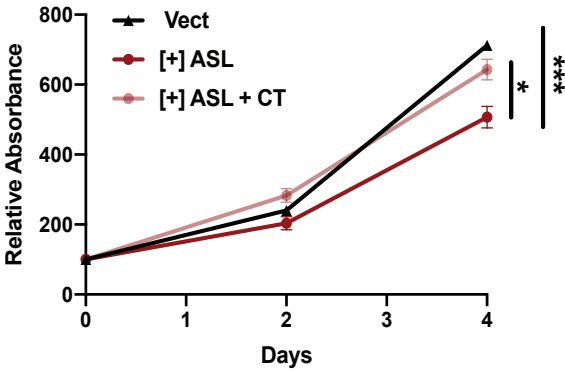

D.

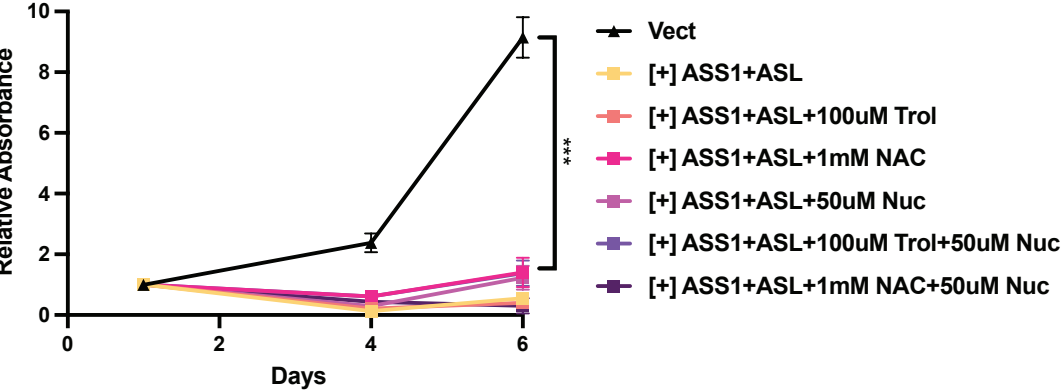

Supp. Figure 3

Supplement: Supplementary file 3 — Additional file 3: Figure S3. ASL Expression in 769-P cells Alters Amino Acid Pools and Affects DNA Replication. A. Steady-state amino acid levels in 769-P cells expressing empty vector and ASL cDNA. Error bars represent SEM of 3 technical replicates. ****p< 0.001, ***p< 0.001. B. Representative images of cell cycle analyses of 769-P cells with propidium iodide (PI) staining indicating a sub-G0 peak in cells expressing ASL cells at 0 hours and 24 hours. C. Exogenously provided pyrimidines (cytidine + thymidine, 50 μM) partially rescue growth in 769-P cells expressing ASL. Error bars represent SEM of 7 wells. *p< 0.05, ***p< 0.001. D. 786-O cells expressing ASS1+ASL cDNA were cultured with Trolox (Trol, 100μM), N-Acetylcysteine (NAC, 1mM), and/or nucleosides (adenosine + guanosine + cytidine + thymidine, 50μM) and assessed for cell proliferation by WST-1. Error bars represent SEM of 7wells. ***p< 0.001. [file 40170_2021_271_MOESM3_ESM.pdf]

**A.**

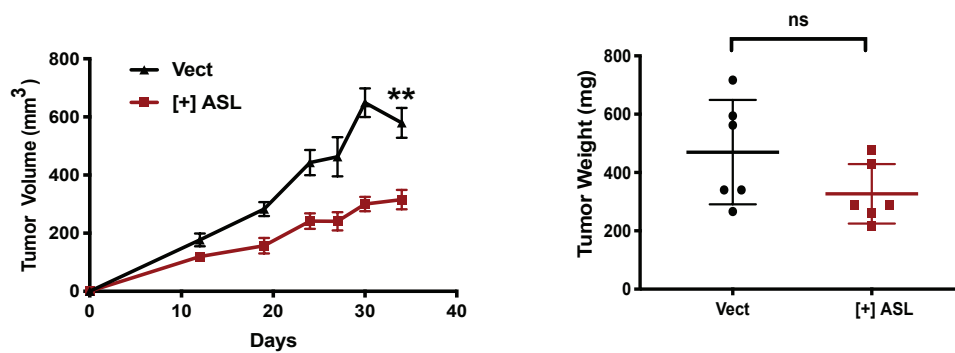

**B.**

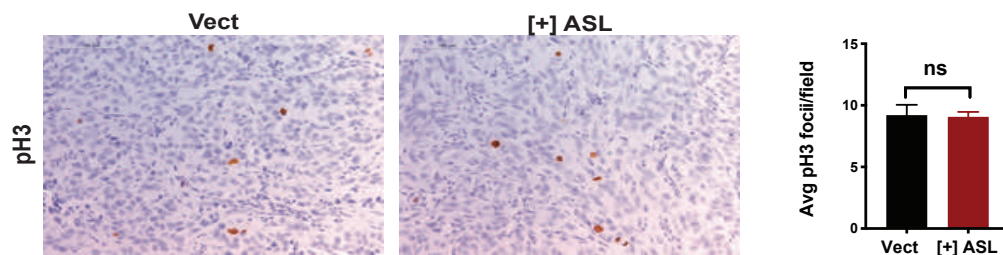

**C.**

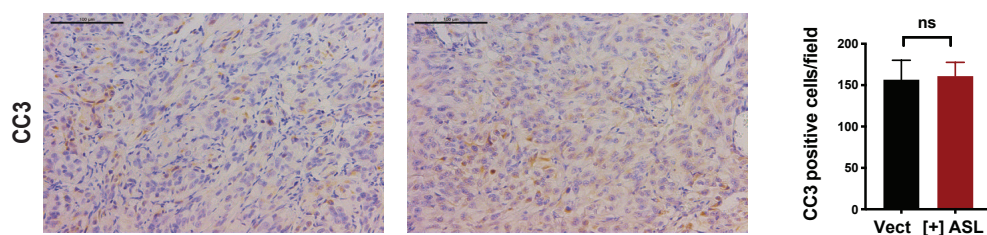

**D.**

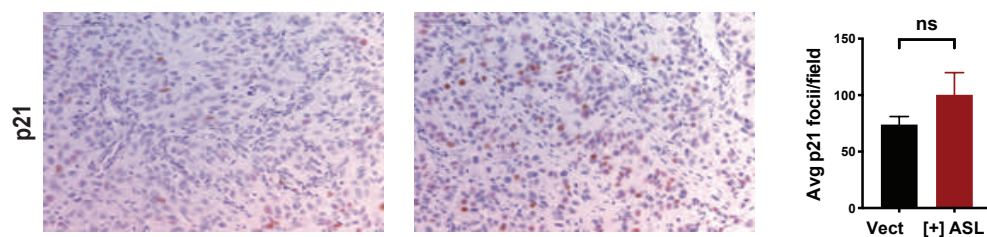

**E.**

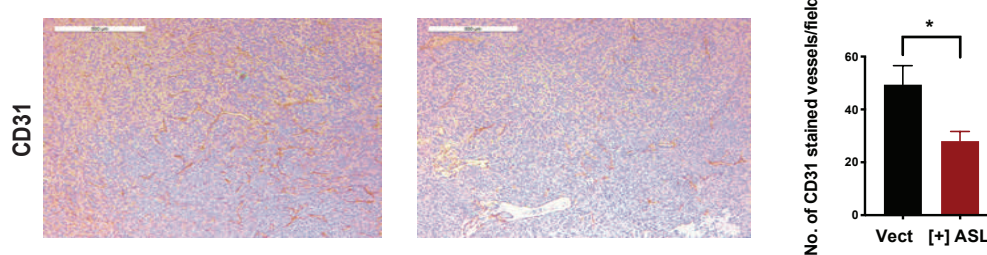

**F.**

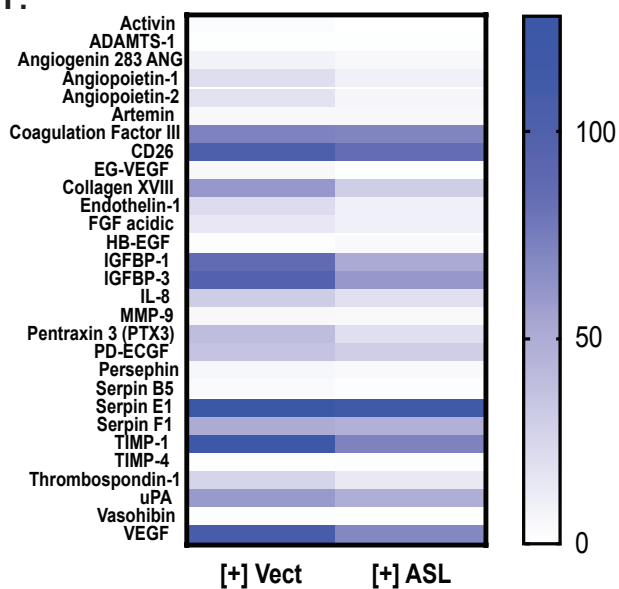

Supp. Figure 4

Supplement: Supplementary file 4 — Additional file 4: Figure S4. Re-expression of ASL Alone in 786-O Cells Does Not Suppress Growth in vivo. A. 786-O subcutaneous xenograft tumor growth in nude mice, where cells expression ASL or empty vector. (n = 5). B. Immunohistochemistry staining and quantification for phosphorylated histone H3 as marker for proliferation in xenograft tumors. Scale bars are at 100μm. C. Immunohistochemistry staining and quantification for cleaved caspase 3 as marker for apoptosis in xenograft tumors. Scale bars are at 100μm. D. Immunohistochemistry staining and quantification for p21 as marker for proliferation in xenograft tumors. Scale bars are at 100μm. E. Immunohistochemistry staining and quantification for CD31 as marker for vasculature in xenograft tumors. Scale bars are at 100μm. *p< 0.05. F. Heat map showing the levels of various angiogenic proteins in 786-O xenograft tumors expressing ASL or empty vector. [file 40170_2021_271_MOESM4_ESM.pdf]
